# Receipts Acknowledged

**Published:** 1867-08

**Authors:** 


#### *Receipts Acknowledged from:*

T. A. Pope, $2; A. H. Scott, $1; J. B. Cox, 1; Henry Warm, 2; C. O'Brien, 2; A. Atwood, 2; H. G. Evers, 2; H. Ziesing, $2; J. W. Trueworths, 2; G. N. Jennings, $1; J. Doron, 2; L. B. Brown, 2; T. T. Linn, 2; N. T. Quales, 2; W. D. Morehouse, 2; J. Blount, 2; H. W. Sigworth, 2; H. Durham, 2; J. Seaverns, 2; C. Amos, 2; Charles Bounce, 2; S. Hawley, 2; J. G. Cameron, 2; G. W. Van Zant, 2; H. C. Horton, 2; D. M. Coole, 2; H. T. Fox, 2; B. F. Trull, 2; W. C. Peatt, 2; H. H. Hooker, 2; R. H. Culbertson, 2; M. Bently, 2; S. L. Tyner, 2; Byron Holmes, 1; J. M. Cowen, 2; A. S. Branch, 2; W. E. Peters, J. A. Goldsborry, 2; Thomas Temple, 2; J. A. Clark, 2; J. Cotton, 2; G. M. Chamberlain, 1; J. N. Morris, 2; W. H. St. Clair, 2; C. A. Barnes, 3; W. H. Bright, 2; G. W. Burns, 2; James McCoy, 6; Charles Booth, 2; H. N. Clark, 2; J. Pratt, 2; Charles Kerr, 2; H. Ludington, 2; J. O'Reilly, 5; W. G. Leonard, 2.

